# Effect of internal limiting membrane surgical techniques on the idiopathic and refractory management of macular holes: a systematic review and meta-analysis

**DOI:** 10.1186/s40942-024-00564-2

**Published:** 2024-06-21

**Authors:** Miguel A. Quiroz-Reyes, Erick A. Quiroz-Gonzalez, Miguel A. Quiroz-Gonzalez, Virgilio Lima-Gomez

**Affiliations:** 1 The Retina Department, Oftalmologia Integral ABC (Medical and Surgical Nonprofit Organization) affiliated with the Postgraduate Studies Division at the National Autonomous University of Mexico, Av. Paseo de Las Palmas 735 Suite 303, Lomas de Chapultepec, 11000 Mexico City, Mexico; 2 Institute of Ophthalmology. (Nonprofit Organization) affiliated with the Postgraduate Studies Division at the National Autonomous University of Mexico, Av. Chimalpopoca 14. Col. Obrera, 06800 Mexico City, Mexico; 3 Juarez Hospital, Public Assistance Institution (Nonprofit Organization), Av. Politecnico Nacional 5160, Colonia Magdalena de Las Salinas, 07760 Mexico, Mexico

**Keywords:** Idiopathic macular hole, Primary macular hole, Refractory macular hole, Tamponade, Internal limiting membrane, Autologous retinal transplantation, Human amniotic membrane grafting

## Abstract

Macular holes (MHs), including atraumatic idiopathic and refractory MHs, affect central vision acuity due to full-thickness defects in the retinal tissue. The existing controversy regarding the pathophysiology and management of MHs has significantly improved with the implementation of internal limiting membrane (ILM) surgical techniques and improved MH closure rates. Thus, to determine the effect of ILM techniques on large idiopathic and refractory MH management, the present study systematically reviewed 5910 original research articles extracted from online literature databases, including PubMed, Cochrane, Google Scholar, and Embase, following the PRISMA guidelines. The primary outcome measures were MH closure rate and postoperative visual acuity. A total of 23 randomized controlled trials (RCTs) with adequate patient information and information on the effect of ILM peeling, inverted ILM flaps, autologous retinal transplantation (ART), and ILM insertion techniques on large idiopathic and refractory MH patients were retrieved and analyzed using RevMan software (version 5.3) provided by the Cochrane Collaboration. Statistical risk of bias analysis was also conducted on the selected sources using RoB2, which showed a low risk of bias in the included studies. A meta-analysis indicated that the inverted ILM flap technique had a significantly greater MH closure rate for primary MH than the other treatment methods (OR = 3. 22, 95% CI 1.34–7.43; p = 0.01). Furthermore, the findings showed that the inverted ILM flap group had significantly better postoperative visual acuity than did the other treatment options for patients with idiopathic MH (WMD = − 0.13; 95% CI = 0.22–0.09; p = 0.0002). The ILM peeling technique had the second highest statistical significance for MH closure rates in patients with idiopathic MH (OR = 2. 72, 95% CI: 1.26–6.32; p = 0.016). In refractory MHs, autologous retinal transplant (ART) and multilayer ILM plug (MIP) techniques improve the closure rate and visual function; human amniotic membrane grafting (hAMG) provides a high degree of anatomical outcomes but disappointing visual results. This study demonstrated the reliability and effectiveness of ILM techniques in improving the functional and anatomical outcomes of large idiopathic and refractory MH surgery. These findings will help clinicians choose the appropriate treatment technique for patients with idiopathic and refractory MH.

**Supplementary Information:**

The online version contains supplementary material available at 10.1186/s40942-024-00564-2.

## Introduction

Macular holes (MHs) are a common cause of retinal disease, particularly those affecting the foveal region, and can lead to significant vision loss. MHs can be divided into different subtypes according to their pathogenesis, morphological characteristics, and therapeutic options [1–3]. MH is morphologically defined as a partial- or full-thickness defect of the neurosensory foveal region due to tangential tractional dehiscence rather than loss or avulsion of the tissue. A full-thickness macular hole (FTMH) extends from the internal limiting membrane to the retinal pigment epithelium (RPE) [1–3]. The reported incidence of MHs is 3.3 per 1000 people [4]. According to researchers, the internal limiting membrane (ILM) plays a major role in MH formation and expansion [5, 6]. Thus, implementing ILM surgical techniques in MH closure can be considered a therapeutic milestone. Statistics show that a 90% closure rate and 80% visual acuity have been successfully achieved in untreatable MHs through treatment with current surgical techniques [3], modern diagnostic tools, and prognostication of individual cases, all of which have improved anatomical and functional outcomes [7].

The predominant and most common subtype of MH is known as primary or idiopathic macular hole (MH) [3], which is mainly age-related in origin and can be a partial-thickness MH (PTMH) or full-thickness macular hole (FTMH) [8]. MH is the main cause of central vision loss and has a high prevalence, especially in elderly female patients aged > 50 years [3, 9, 10]. The pathogenesis of primary MH is still unclear [10]. The gradually occurring clinical manifestations of idiopathic MH include decreased vision, difficulty reading when the condition is bilateral, metamorphopsia, and central dark spots [11]. ILM peeling has been defined as the primary treatment for idiopathic MH [9]. Furthermore, the molecular status of the vitreous substitute should include all the structural and functional qualities of the physiological vitreous. Vitreous substitutes, such as air, sulfur hexafluoride (SF_6_), perfluoroethane (C_2_F_6_), perfluoropropane (C_3_F_8_), and silicone oil (SO), can be classified based on their function or molecular status to provide postoperative tamponade (SO).

Refractory MH, another type of full-thickness MH (FTMH), is challenging for clinicians because these holes cannot be closed or reopened after a complete primary surgery. Moreover, various new and innovative techniques have been proposed for refractory MHs [1]. However, with novel surgical modalities, a minimal percentage of MHs still have a greater risk of primary surgical failure. In this form, in chronic large primary and refractory MH, several reports have shown that modern techniques, including ILM flap manipulations in combination with surgical adjuncts, increase anatomical closure success but still result in disappointing visual outcomes; examples include medium or mainly large refractory MHs without central ILMs where surgical options such as pedicle ILM flaps, retracting ILM doors, ILM insertion, autologous free ILM flaps, ILM distal flaps, enlarged ILM peeling, autologous retinal grafting (ARG) or autologous retinal transplant (ART), human amniotic membrane grafting (hAMG), multilayer internal limiting membrane plug (MIP), adjuvant chorioretinal adhesives, and experimental mesenchymal stem cells in experimental assays, all of which have proven to be beneficial in the anatomical closure of these challenging MHs [3–5]. Secondary MHs are associated with pathologic myopia, eye trauma, proliferative diabetic retinopathy, and other vitreoretinal conditions [3]. In addition to these techniques, various treatments such as gas type, tamponade, posturing, ocriplasmin, and 27-gauge microincision vitrectomy surgery (MIVS) have been used to treat MHs, and both successful and failed visual gain and anatomical closure have been observed [4].

Management of MHs has evolved from an untreatable condition to a microsurgical procedure with considerable potential success [10], where the rate of visual acuity represents successful MH surgery [4]. Thus, the present study was designed to review the applications and successful effects of ILM surgical techniques in patients with large idiopathic and refractory MH closure, as well as anatomical and functional outcomes and visual acuity improvements after treatment with ILM techniques.

## Materials and methods

### Article collection

1. A systematic review of the literature was conducted in accordance with the Preferred Reporting Items for Systematic Reviews and Meta-Analyses (PRISMA) guidelines. The research articles were searched in publicly available online literature databases, including PubMed (https://pubmed.ncbi.nlm.nih.gov/), Scopus (https://www.scopus.com/home.uri), Ovid (https://ovidsp.ovid.com/), Cochrane (https://www.cochranelibrary.com/), Google Scholar (https://scholar.google.com/), and Embase (https://www.elsevier.com/en-in/solutions/embase-biomedical-research), using Medical Subject Headings (MeSH) terminologies such as “Idiopathic large macular hole AND refractory macular hole AND internal limiting membrane techniques AND surgery,” “Idiopathic macular hole AND refractory macular hole AND internal limiting membrane removal technique,” “Idiopathic macular hole AND refractory macular hole AND lens capsule transplantation,” “Idiopathic macular hole AND refractory macular hole AND inverted internal limiting membrane technique,” “Idiopathic macular hole AND refractory macular hole AND inverted internal limiting membrane technique,” “Idiopathic macular hole AND free autologous limiting membrane transplantation,” “Idiopathic macular hole AND refractory macular hole AND autologous neurosensorial retinal grafting,” “Idiopathic macular hole AND refractory macular hole AND amniotic membrane grafting technique,” “Idiopathic macular hole AND refractory macular hole AND amniotic membrane grafting technique,” and “Idiopathic macular hole AND refractory macular hole AND amniotic membrane grafting technique,” and “Idiopathic macular hole AND refractory macular hole”. “Idiopathic macular hole AND refractory macular hole AND FSIP technique AND FS-ILM removal technique”. The articles were screened according to the inclusion and exclusion criteria (see the search strategy in Appendix 1 in the Supplementary file).

### Inclusion criteria

2. Articles with complete information on the anatomical and functional effects of diverse ILM techniques in large idiopathic and refractory MH surgeries.
3. Articles should be written in the English language.
4. The full length of the original research articles should be available.
5. The studies included only human samples.
6. Only randomized controlled trials (RCTs) were considered for further statistical analysis.
7. Studies could have been performed in any country.

### Exclusion criteria

1. Review articles, letters to the editor, discussions, single case reports, meta-analyses, abstracts, systematic reviews, and articles published in other languages
2. Articles with unwanted data, such as other diseases or treatment methods.
3. Articles with missing information on treatment methods, patient information, and mortality rate.
4. Nonrandomized studies.
5. Nonhuman studies were also excluded.
6. Studies pertaining to age-related macular degeneration or other diagnoses unrelated to idiopathic primary or refractory MHs were excluded.

### Article screening process

Articles collected via database searches using MeSH terms were imported into Covidence.org. Duplicate studies were removed, and systematic screening was conducted by two authors (MAQR and EAQG). Titles and abstracts were screened, and KAPPA statistics were computed for each filtering stage before discrepancies were resolved. In the event of disagreement, a third reviewer (VLG) was consulted for resolution. The complete texts of the eligible studies were uploaded for full screening. Again, the KAPPA statistics were computed before discrepancies were resolved. The following information was extracted from all studies: (1) general information about the purpose of the study, aim, and outcomes; (2) protocol methodology using the study design, inclusion and entry criteria, study participants, methods, and follow-up period; (3) visual acuity before and after treatment; (4) type of ILM removal technique used at the time of surgery; and (5) safety outcomes and complications during and after diverse ILM removal or manipulation techniques.

The filtered articles were again screened based on the inclusion and exclusion criteria. Articles with the required information, particularly concerning the effects of ILM techniques on large idiopathic and refractory MH surgeries were considered for further analysis.

### Data retrieval

The collected articles were screened manually, and the required data were retrieved by two independent authors (MAQR and EAQG), including the authors’ information, principal author’s last name, publication year, PubMed IDs, study groups, study design, sample size (number of studied idiopathic primary or refractory MH cases), mean MH size and visual acuity, study region, participant characteristics (mean age and sex), treatment methods used, number of patients treated with different ILM techniques, MH closure rates, pre- and postoperative best-corrected visual acuity (BCVA), and proof of informed consent. The retrieved data were analyzed according to the PICOS format as follows:

P: population: number of patients with large idiopathic or refractory MHs.

I: intervention: number of MH patients treated with ILM techniques.

C: comparator: number of patients treated with techniques other than the ILM technique.

O: outcome: the effect of ILM techniques on large idiopathic or refractory MH patients.

S: private and hospital settings.

### Risk of *Bias* analysis

The retrieved data were analyzed using the Cochrane risk of bias tool for randomized trials (RoB 2) in R. The risk of bias analysis was based on five possible domains: (D1) bias arising from the randomization process, (D2) bias due to deviations from intended interventions, (D3) bias due to missing outcome data, (D4) bias in the measurement of the outcome, and (D5) bias in the selection of the reported result. The inputs under each domain led to the generation of graphical representations of “low risk of bias,” “some concerns,” or “high risk of bias” [12].

### *Meta*-analysis

RevMan 5.3 software provided by the Cochrane Collaboration was used for the statistical analysis. Standard deviations (SDs) and means were used to calculate the weighted mean differences with 95% confidence intervals (CIs). Odds ratios (ORs) with 95% CIs were also calculated. The X2 test was used to assess the statistical heterogeneity between the studies included in the analysis. For P < 0.05 and I2 > 50, heterogeneity was considered significant, and a random effect model was adopted. However, in cases where I2 was ≤ 50%, heterogeneity was considered low, and the fixed-effects model was used for data analysis.

## Results

### Study selection

**Fig. 1:**
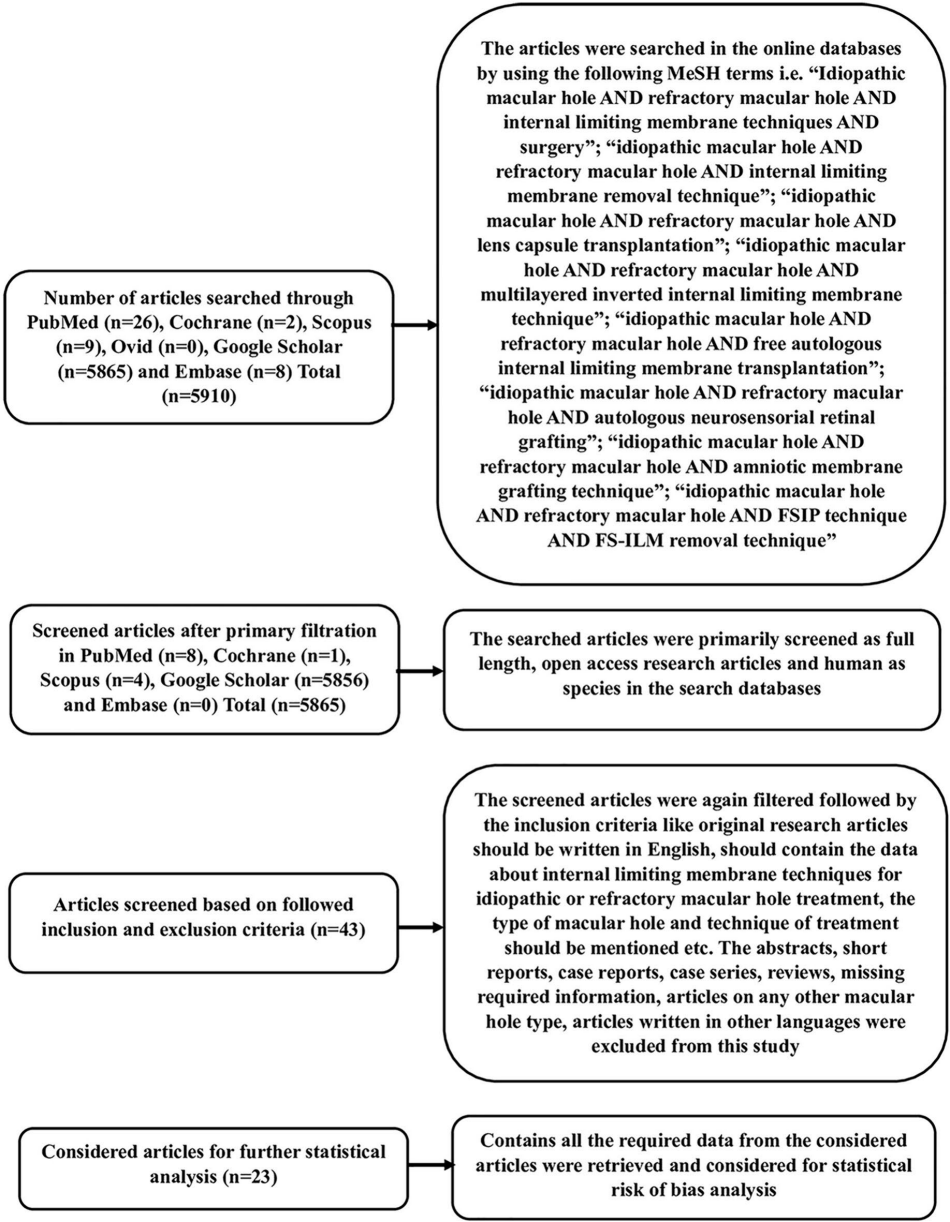
PRISMA flow diagram of the systematic review

A total of 5910 articles were identified in five online repositories: PubMed (26), Scopus (9), Cochrane (2), Google Scholar (5865), and Embase (8). No results were obtained from the Ovid literature database based on the medical subjet heading (MeSH) terms used. Among these search results, only 5865 articles were further considered after identifying only open-access articles with research conducted on human species in all five databases, and the researchers included open-access articles. The articles were screened again by applying the inclusion and exclusion criteria, and only 34 studies were found that contained all required and PICOS data. However, among these studies, only 23 were RCTs and were subjected to further risk-of-bias analysis. The study selection was performed using the PRISMA flow diagram (Fig. 1).

### Study characteristics

The included RCTs were published between 2013 and 2023. A total of 721 patients with MH were included: 448 had idiopathic MHs, and the remaining 273 had refractory MHs. The treatment techniques included ILM peeling, the inverted ILM flap technique, ART, and ILM insertion. ILM peeling and the inverted ILM flap technique were applied in primary large MHs, while ART and ILM insertion were used in refractory MHs. A total of 23 RCTs with 340 eyes in the inverted ILM flap group, 392 eyes in the ILM peeling group, 401 eyes in the ART group, and 309 eyes in the ILM insertion group were included in this meta-analysis. The ages of the patients ranged from 20 to 85 years. The geographic locations of the studies were multicenter and included China, Brazil, Canada, Italy, Japan, the United States, India, Argentina, Taiwan, Egypt, Greece, Thailand, Iran, Pakistan, Germany, and Tunisia (Table 1).

**Table 1:** Retrieval characteristics with visual acuity differences in all included studies

Sl. no	Authors & PubMed IDs	No. of patients	Study setting	Age	Clinical diagnosis	Anatomical closure rates	Used treatment method	Pre-post BCVA (logMAR) and output	References
1	Peng et al., 2020 (PMID:32420222)	26	China	45–75	IMH	87.5%	ILM dragging and peeling	VA improved from 1.2 ± 0.6 to 0.7 ± 0.5 (*p* < 0.001). It improves the closure rate of large IMH	[34]
2	Sinawat et al., 2021 (PMID:33159177)	122	Thailand	Mean age 62.37 ± 4.43	IMH	91.3%	Classic ILM peeling	VA improved from 1.25 ± 0.37 to 0.39 ± 0.43 *(p* < *0.001).* It yielded a significantly higher closure rate	[35]
3	Kumagai et al., 2013 (PMID:23696603)	24	Japan	Mean age 65	IMH	67.4%	Classic ILM peeling	VA improved from 0.84 to 0.23 at 6 months (p < 0.001). It’s a successful treatment procedure	[36]
4	Zong et al., 2021 (PMID:34608417)	16	China	48–72	IMH	72.8%	ILM and flap inverting under air	VA improved from 1.49 ± 0.35 to 0.89 ± 0.35, and visual acuity of 20/100 or better was achieved in 8 eyes. This method was helpful in improving the functional and anatomic outcomes in IMH	[37]
5	Huang et al., 2020 (PMID:32703197)	22	China	Mean age 62	IMH	85.1%	Free ILM flap tamponade technique	Mean VA improved from 1.10 ± 0.33 to 0.67 ± 0.32 at 6 months postoperatively (*p* < 0.001). Resulted effective morphological and functional recovery for large IMHs	[38]
6	Zgolli H et al., 2020 (https://doi.org/10.32512/jmr.3.3.2020/3.8)	36	Tunisia, North Africa	Mean age 57 ± 4 58 ± 8 65 ± 4 & 64	IMH	64.8%	Inverted ILM flap techniques	In group 1, the mean BCVA improved from 1.25 ± 0.12 to 1.15 ± 1.16 at 3 months (*p* = 0.8). In group 2, BCVA improved from 0.69 ± 0.42 to 0.27 ± 0.16 at 3 months (*p* = 0.086). In group 3, BCVA improved from 0.69 ± 0.44 to 0.27 ± 0.16 at 3 months (*p* = 0.086). In the fourth group, BCVA improved from 1 ± 0.32 to 0.38 ± 0.2 at 3 months (*p* = 0.236). It is an effective treatment for large FTMH	[39]
7	Zhang et al., 2023 (PMID:36844949)	13	China	Mean age 65.769 ± 3.479	IMH	62.9%	Inverted ILM flap technique	VA improved from 1.208 ± 0.158 to 0.708 ± 0.131 at 6 months. This is an effective treatment and can reconstruct macular anatomical structure and improved VA	[40]
8	Nowroozzadeh et al., 2018 (PMID:29719640)	10	Iran	56–78	IMH	85.2%	Free ILM flap transplantation	Mean VA improved from 1.35 ± 0.32 to 0.78 ± 0.37 (*p* < 0.001). It resulted in high anatomic closure and improvement in EZD	[41]
9	Hirata et al., 2021 (PMID:34625615)	13	China	Mean age 68.3 ± 5.9	IMH	76.7%	Temporal ILM flap	Mean BCVA improved from 0.63 ± 0.37 to 0.18 ± 0.15 (*p* < 0.001). It confers a good visual outcome	[42]
10	Tayyab et al., 2019 (PMID:31086507)	11	Pakistan	63.41 ± 5.93	IMH	70.3%	Inverted ILM flap technique	Mean BCVA improved from 1.236 ± 0.265 to 0.918 ± 0.336 months (*p* < 0.05). Its an effective method for repairing large MHs	[43]
11	Bleidibel et al., 2021 (PMID:33512612)	54	Germany	Mean age 67 ± 7	Idiopathic FTMH	87.5%	Inverted ILM flap	Mean BCVA improved from 0.98 ± 0.38 to 0.42 ± 0.33 at 12 months (*p* < 0.001). Favorable morphological and functional outcomes observed	[44]
12	Carpineto et al., 2021 (PMID:33628475)	16	China	59–78	Idiopathic FTMH	91.3%	Inverted ILM flap	Mean BCVA improved from 1.1 to 0.3 at 6 months. This is an effective and safe technique	[45]
13	Moysidis et al., 2021 (PMID:33045315)	130	Multicentric	Mean 63 years	RMH	89.7%	ART	VA improved from 1.37 ± 0.12 to 1.05 ± 0.09 (*p* < 0.001) at 8.6 ± 0.8 months. Patients under ART achieved good anatomic and functional outcomes with low complications rate	[46]
14	Ferreira et al., 2021 (PMID:33964971)	19	Brazil, Canada	23–85	RMH	79.7%	hAMG	Median BCVA improved from 1.30 ± 0.44 (0.80–2.0) to 1.0 ± 0.72 (*p* < 0.0001). This treatment can be a viable and effective alternative for the treatment of large and persistent MHs	[47]
15	Grewal et al., 2019 (PMID:30711606)	41	Italy, Japan, The United States	63–69	RMH	80.4%	ART	Mean VA improved from 1.11 ± 0.66 to 1.03 ± 0.51 *(p* = 0.03) at mean follow-up of 11.1 ± 7.7 months. ART offers high degree of anatomic development and safe for refractory MHs closure	[48]
16	Srivastava et al., 2022 (PMID:36349167)	10	India	58–72	IMH	89.8%	AMP	BCVA improved from 0.91 ± 0.11 to 0.28 ± 0.06 at 3 months. This method is an effective method to treat and manage idiopathic MH with better results in anatomical closure and VA gain	[49]
17	Alezzandrine et al., (PMID:34600572)	28	Argentina	–	RMH	82.6%	ART	In AT-ILM group, BCVA improved from 0.90 (Snellen 20/160) to 0.70 (Snellen 20/100) *(p* = 0.006). It results in better visual outcomes	[50]
18	Wu et al., 2018 (PMID:30157808)	6	Taiwan	41–68	RMH	79.61%	ARG & ABC	VA improved from 1.47 ± 0.31 to 1.09 ± 0.52 at 12 months. These surgical techniques provide an option for the treatment of refractory MH	[51]
19	Lee et al., 2022 (PMID:35586596)	9	Taiwan	40–75	RMH	81.3%	ART	Mean BCVA improved from 1.61 ± 0.44 to 0.72 ± 0.30 at 12 months (*p* < 0.001). This treatment provides long-term good anatomical and functional results	[52]
20	Yuan et al., 2020 (PMID:32801616)	40	China	Mean age 55–58	RMH	85.9%	Autologous ILM transplantation	BCVA improved from 1.52 ± 0.29 to 1.09 ± 0.33 at 3 months. The VA of patients improved significantly improving their vision-related QoL	[53]
21	Zhang et al., 2019 (PMID:31823798)	18	China	Mean age 63.67	Idiopathic FTMH	82.6%	ILM insertion	BCVA improved at 3rd and 6th months in the NGF group, and in the insertion group after 6 months *(p* = 0.0081, 0.0276, and 0.0255, respectively, compared to baseline). The NGF group exhibited better recovery than the insertion group at 3rd month follow-up (*p* = 0.0301). It is an effective technique for the initial surgical treatment of eyes with large MHs	[54]
22	Ma et al., 2019 (PMID:31131248)	42	China	54–76	IMH	80.1%	Free autologous ILM transplantation	Mean BCVA improved from 1.37 ± 0.05 to 1.03 ± 0.06 at 1 month (*p* = 0.0001), 0.802 ± 0.045 at 3 months (*p* = 0.011), 0.67 ± 0.04 at 6 months *p* = 0.048) and 0.51 ± 0.06 at 12 month (*p* = 0.034). (*p* < 0.001). Effective anatomical and functional improvement for the treatment	[55]
23	Rayes et al., 2022 (PMID:36376951)	15	Egypt & Greece	20–60	Idiopathic FTMH	95.3%	MIP	Mean BCVA improved from 1.20 (0.06 decimal units (range 0.01–0.1) to 0.70 (0.2 decimal units (range 0.05–0.5). Its effective in promoting macular hole closure and visual function	[56]

ABC: autologous blood clot, AMP: amniotic membrane plug, BCVA: best-corrected visual acuity, hAMG: human amniotic membrane graft, ARG: autologous retinal graft, ART: autologous retinal transplant, EZD: ellipsoid zone density, FTMH: full-thickness macular hole, ILM: internal limiting membrane, IMH: idiopathic macular hole, logMAR: logarithm of the minimum angle of resolution, MIP: multilayer ILM plug, MHs: macular holes, PMID: PubMed identifier, RMH: refractory macular hole, VA: visual acuity

All the patients included in the study were diagnosed with either primary or refractory MHs. Only MH patients (with both primary and refractory MH) with a minimum diameter greater than 400 µm were included in the study. A minimum follow-up period of 6 months was used for all patients. None of the patients included in the meta-analysis underwent any other retinal surgery, either before or after the surgical intervention, to treat idiopathic or refractory conditions.

According to these studies, the inverted ILM flap, which is the most commonly used technique, has been used in most primary MH cases and is considered safe and effective for repairing large FTMHs. These studies also indicate that this technique is mainly used for the treatment of large MHs but is not used for refractory MHs. This approach resulted in favorable morphological and functional outcomes and improved visual acuity in patients with these conditions.

Classical ILM peeling was the second most commonly used technique in patients with large idiopathic MH in the studies considered in this analysis. It has been reported that ILM peeling is helpful for improving anatomical and functional outcomes but yields a significantly lower MH closure rate than does an inverted ILM flap.

Patients with refractory MHs achieved good and high degrees of anatomical and functional outcomes with low complication rates and high MH closure rates using ART. In addition to these techniques, the remaining ILM techniques, including the hAMG and MIP, can be used as good treatment options, as they have also provided better outcomes, including an improved closure rate and visual function in patients with large idiopathic and refractory MHs.

### Statistical analyses

#### Risk of *Bias* analysis

**Fig. 2:**
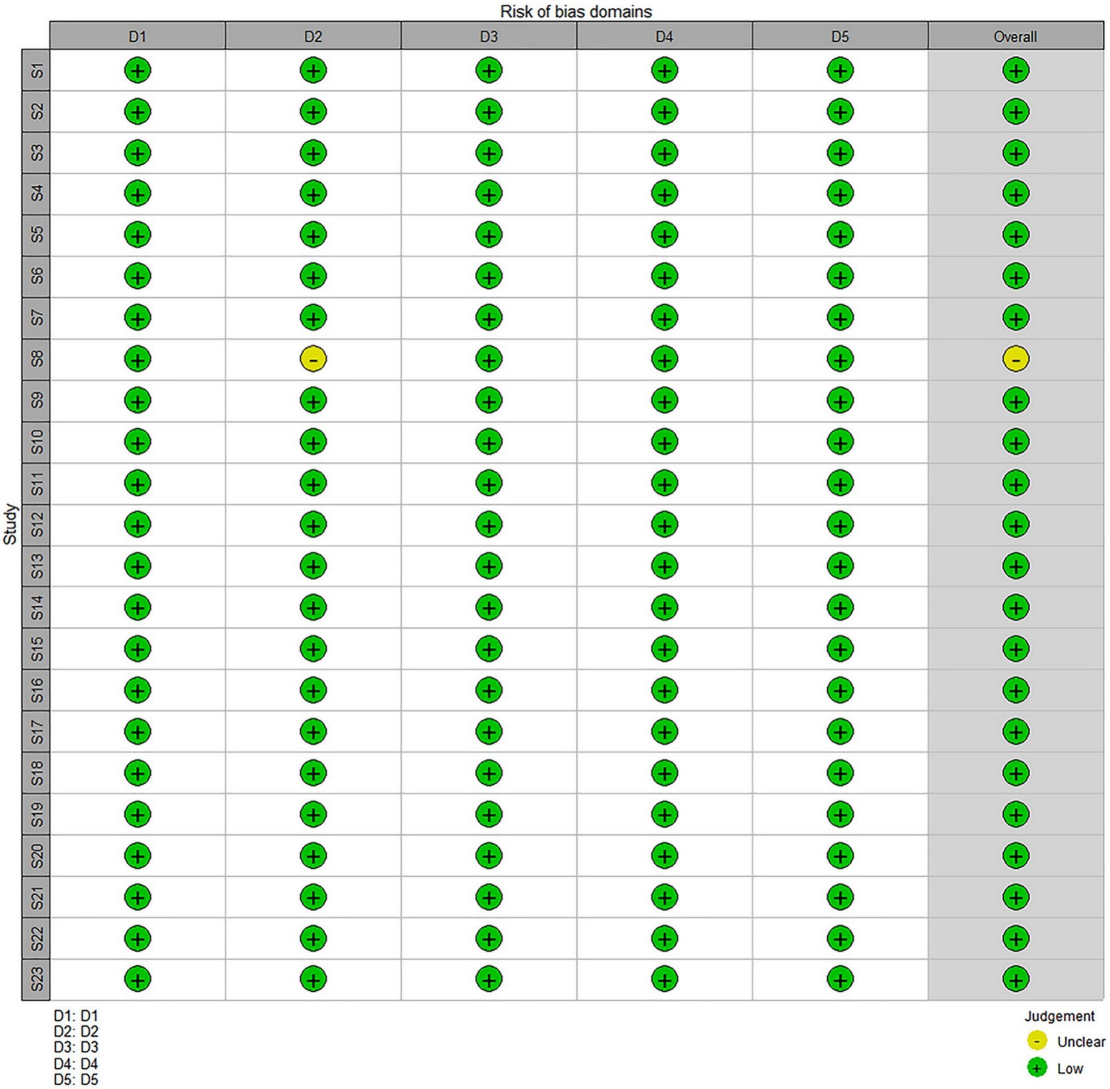
Graphical representation of the risk of bias analysis of individual studies. A low risk of bias was shown in all 23 studies

**Fig. 3:**
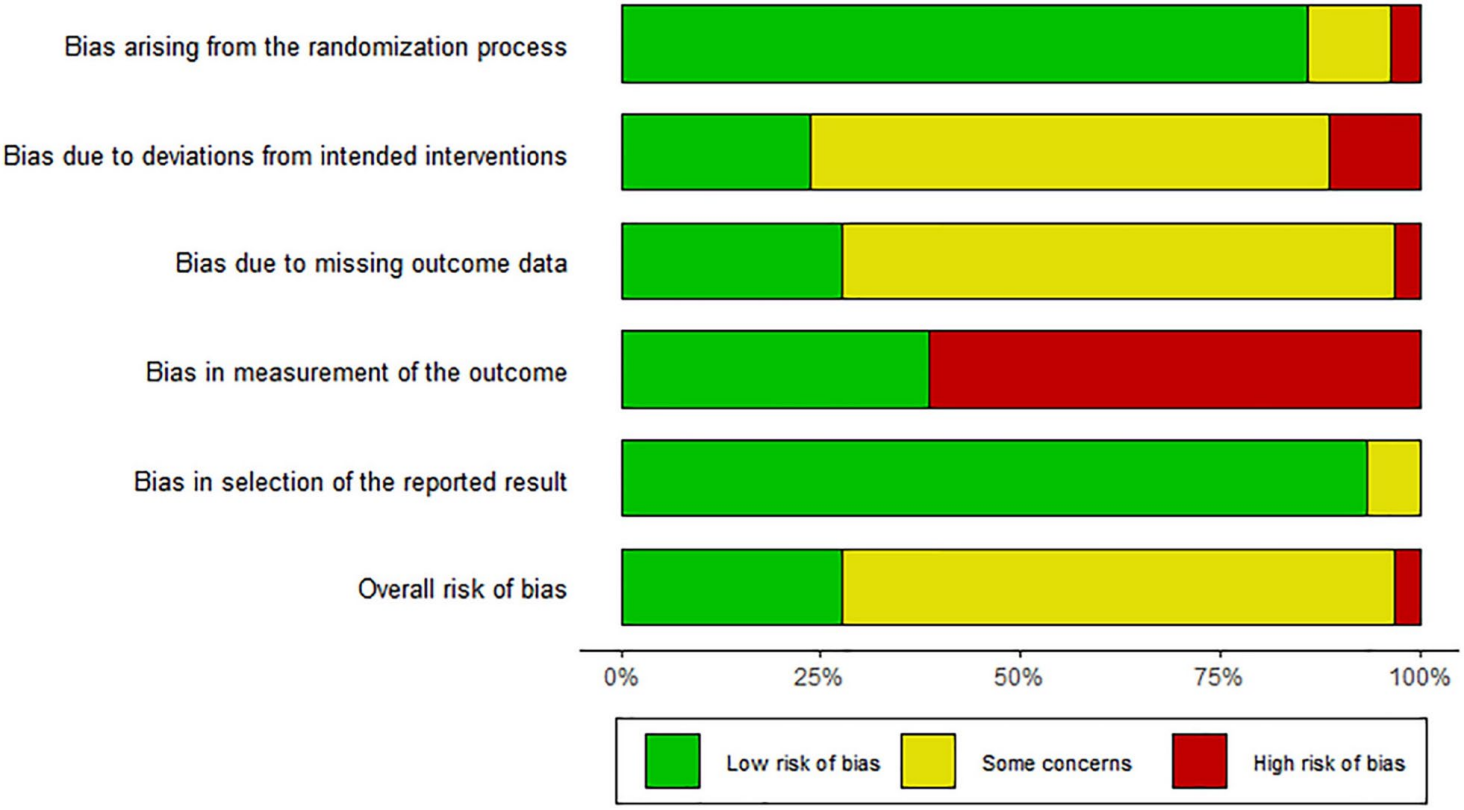
Schematic representation of the summary of the risk of bias analysis. Plot showing an overall low risk of bias

All observed data from the considered articles were subjected to risk of bias analysis. A risk of bias analysis was performed for each study, which predicted a low risk of bias in all twenty-three studies (Fig. 2). Only Study 8 showed an unclear risk of bias in domain 2 (D2) owing to missing information regarding the intended interventions. The overall study bias data are represented in the risk of bias plot with almost 70% having some concerns (Fig. 3), which includes “low risk of bias,” “some concerns for bias” and “high risk of bias,” represented by green, yellow, and red, respectively. However, the overall bias was low.

#### Outcomes of the meta-analysis

**Fig. 4:**
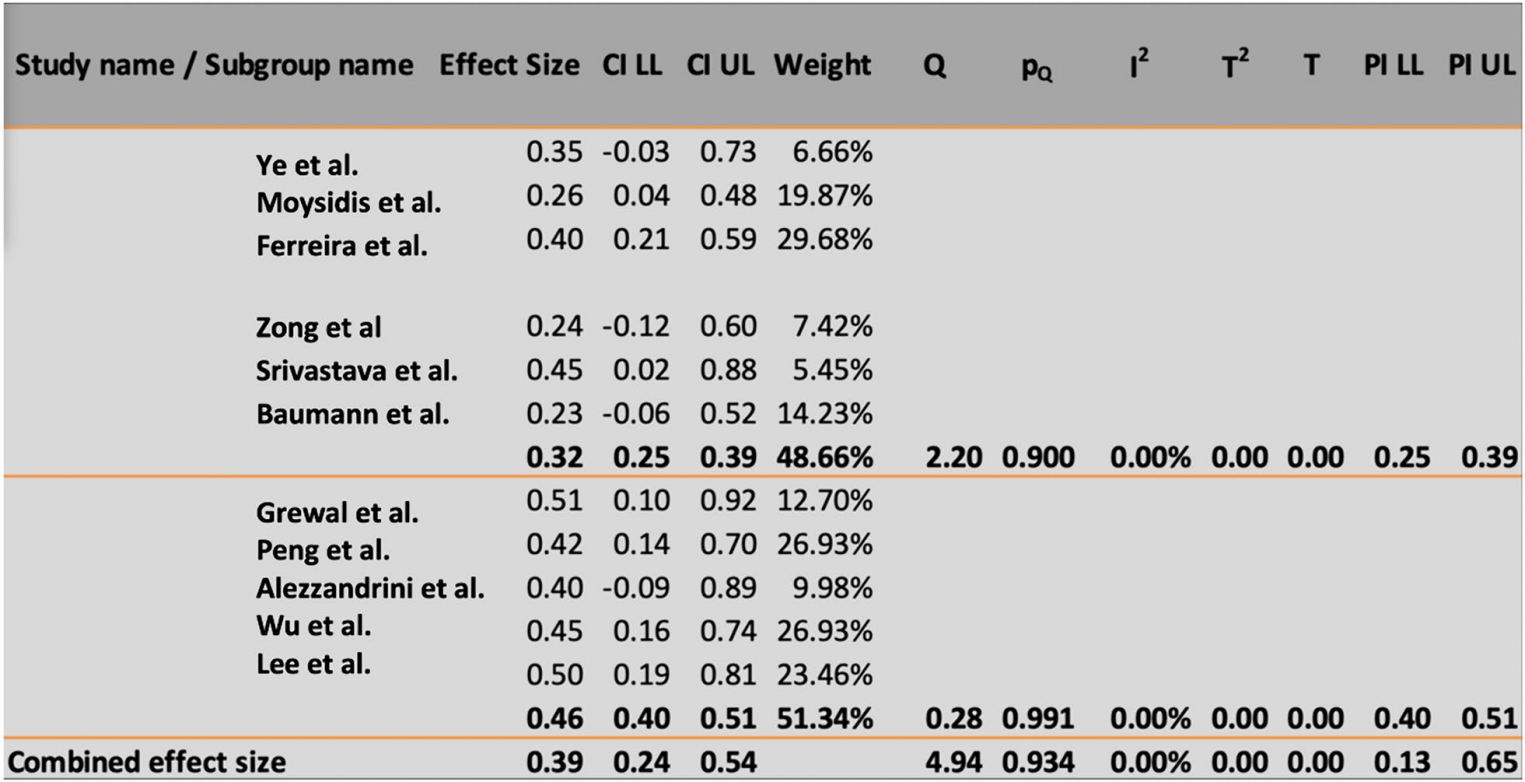
Subgroup analysis of the included studies (MH closure rate). CI: confidence interval, LL: lower limit, UL: upper limit, I^2^: percentage of variation across studies due to heterogeneity rather than chance, Q: adjusted p values, T^2^: difference between the mean values of two the groups, T: t value measures the size of the difference relative to the variation in sample data

**Fig. 5:**
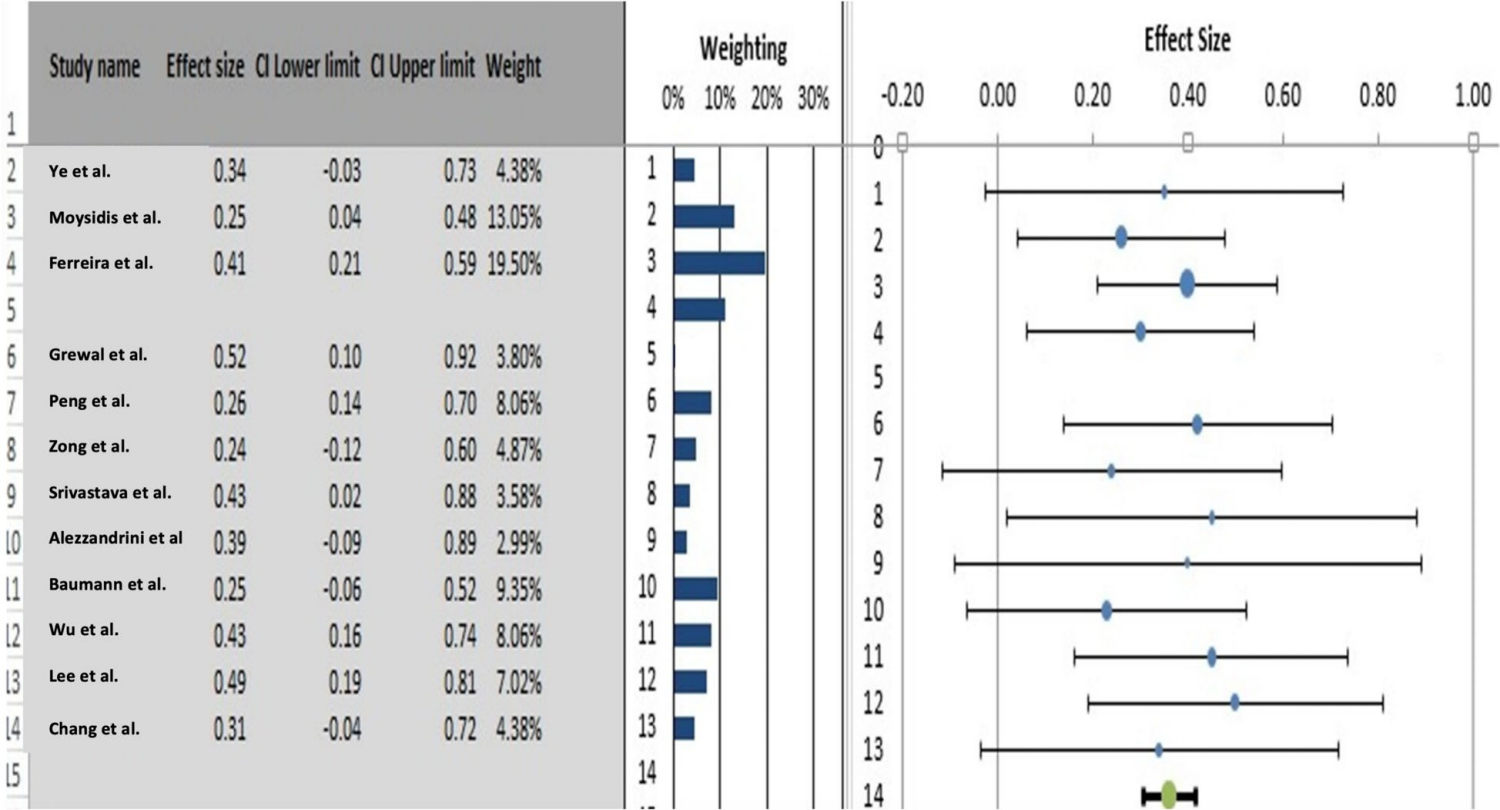
Forecast plot of the included studies (MH closure rate). CI: Confidence interval

**Fig. 6:**
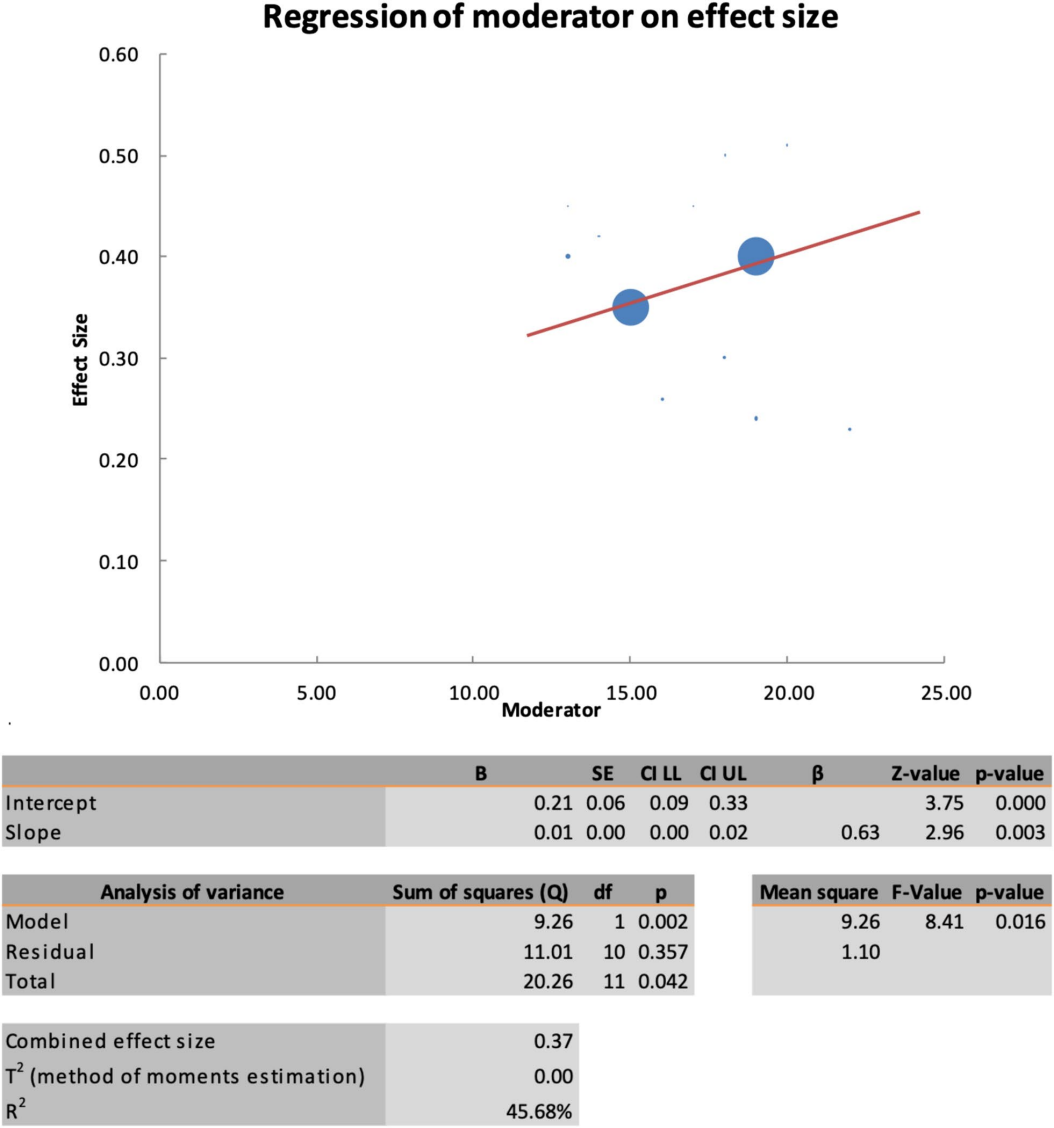
Regression of the moderator on effect size (MH closure rate). SE: standard error, LL: lower limit, UL: upper limit, T^2^: difference between the mean values of the two groups, R^2^: coefficient of determination

The primary outcome measures were the MH closure rate and postoperative visual acuity improvements (anatomical and functional outcomes). The overall MH closure rate was compared among the four treatment techniques, namely, ILM peeling, the inverted ILM flap technique, ART, and ILM insertion, across 12 studies. As no statistical heterogeneity was found (I^2^ = 0%, as shown in Figs. 4 and 5), the fixed-effects model was used for data analysis. The study findings indicated that the inverted ILM flap group had a significantly greater MH closure rate for idiopathic MH than did the other treatment groups (OR = 3. 22, 95% CI 1.34–7.43; p = 0.002, as shown in Fig. 6). The ILM peeling technique had the second highest statistical significance for MH closure rates in idiopathic MH patients (OR = 2. 72, 95% CI 1.26–6.32; p = 0.016). ART and ILM insertion had the least success in enhancing MH closure in patients with refractory MH.

**Fig. 7:**
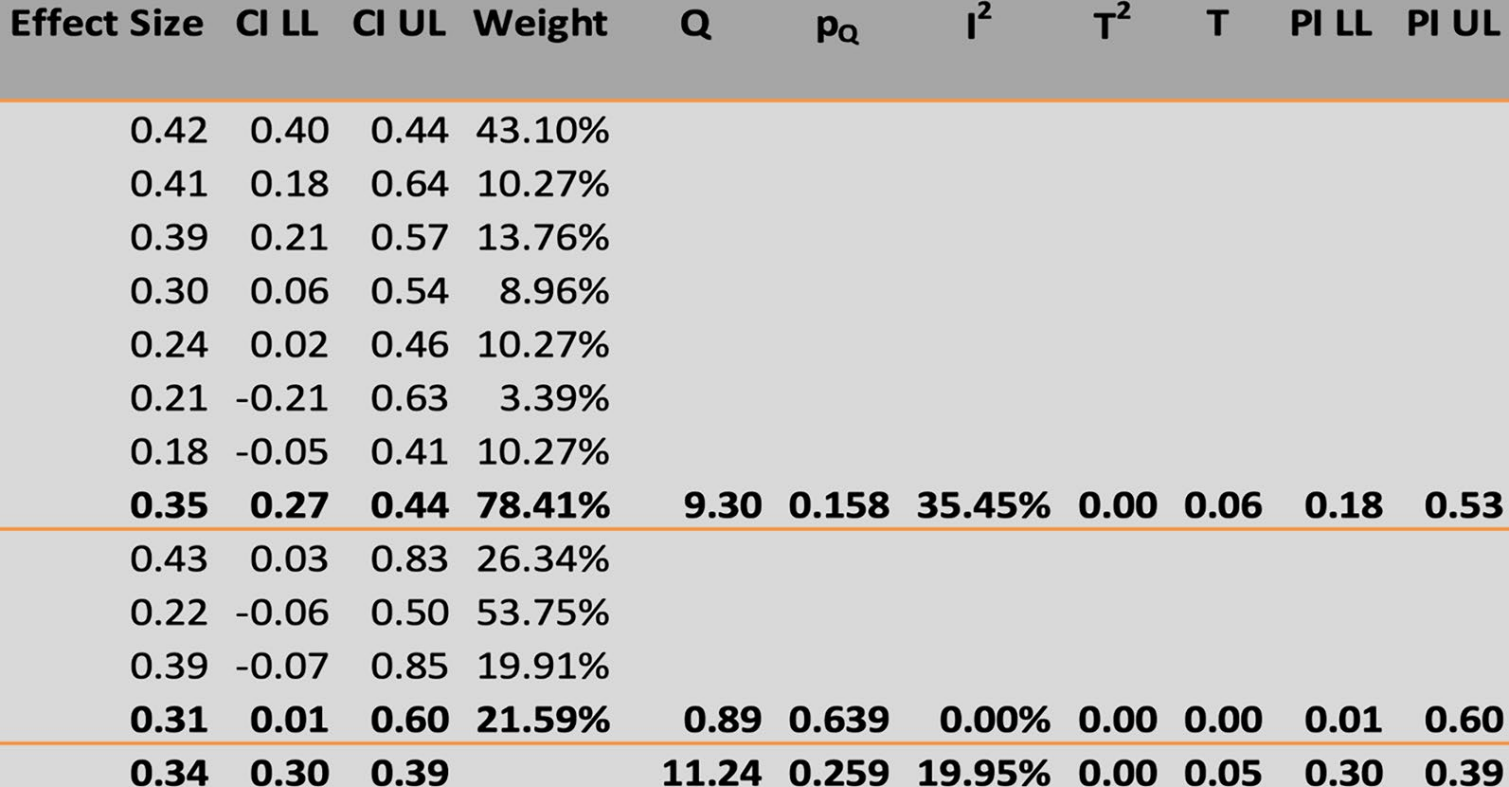
Subgroup analysis of the included studies (preoperative visual acuity). CI: confidence interval, LL: lower limit, UL: upper limit, I^2^: percentage of variation across studies due to heterogeneity rather than chance, Q: adjusted p values, T^2^: difference between the mean values of the two groups, T: t value measures the size of the difference relative to the variation in sample data

**Fig. 8:**
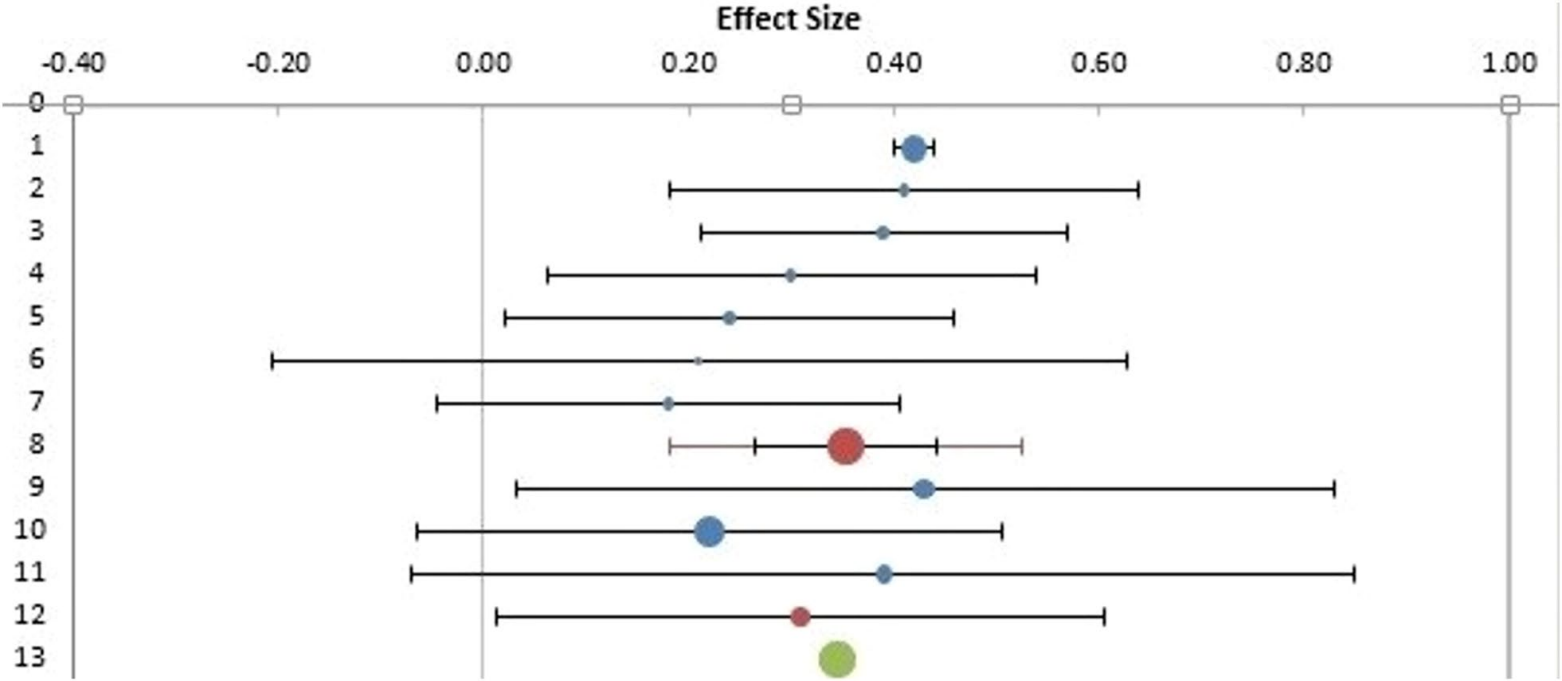
Forecast plot of the included studies (preoperative visual acuity)

**Fig. 9:**
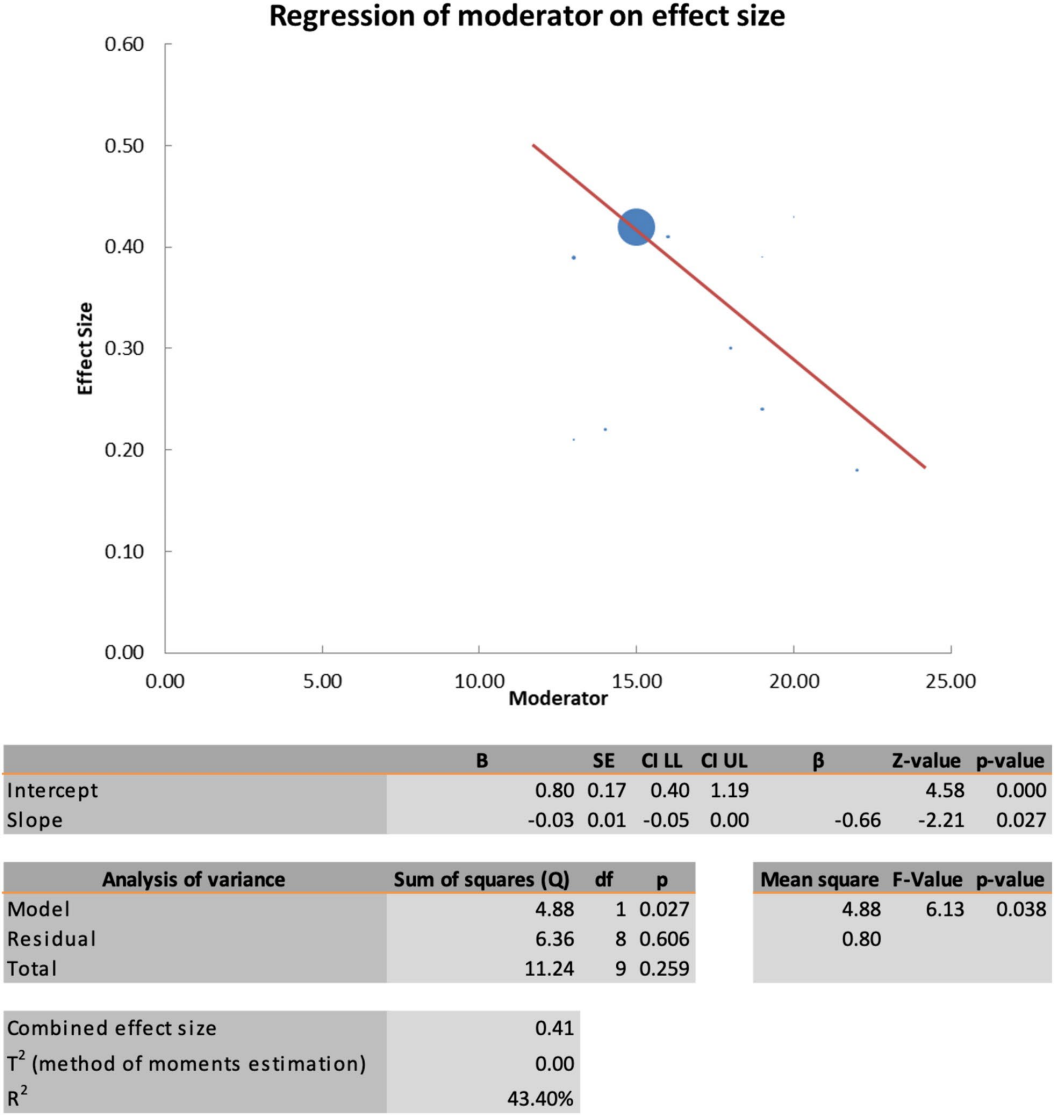
Regression of the moderator on effect size (preoperative visual acuity). SE: standard error, LL: lower limit, UL: upper limit, T^2^: difference between the mean values of the two groups, R^2^: coefficient of determination

Ten studies were pooled to compare preoperative visual acuity among the four treatment techniques. In this study, preoperative visual acuity was used as a measure of functional and anatomical outcomes in patients who underwent idiopathic or refractory MH surgery. As no significant heterogeneity was found (I^2^ = 35.45%, as shown in Figs. 7 and 8), a fixed-effects model was adopted for the data analysis. The findings of the meta-analysis indicated that the inverted ILM flap group had significantly better postoperative visual acuity than the other treatment options for idiopathic MH patients (weighted mean difference (WMD) = − 0.13; 95% CI = − 0.22 − − 0.09; p = 0.0027, as shown in Fig. 9). The ILM peeling technique had the second-highest statistical significance with regard to postoperative visual acuity in idiopathic MH patients (WMD = -0.10; 95% CI = − 0.18, − 0.06; p = 0.038).

**Fig. 10:**
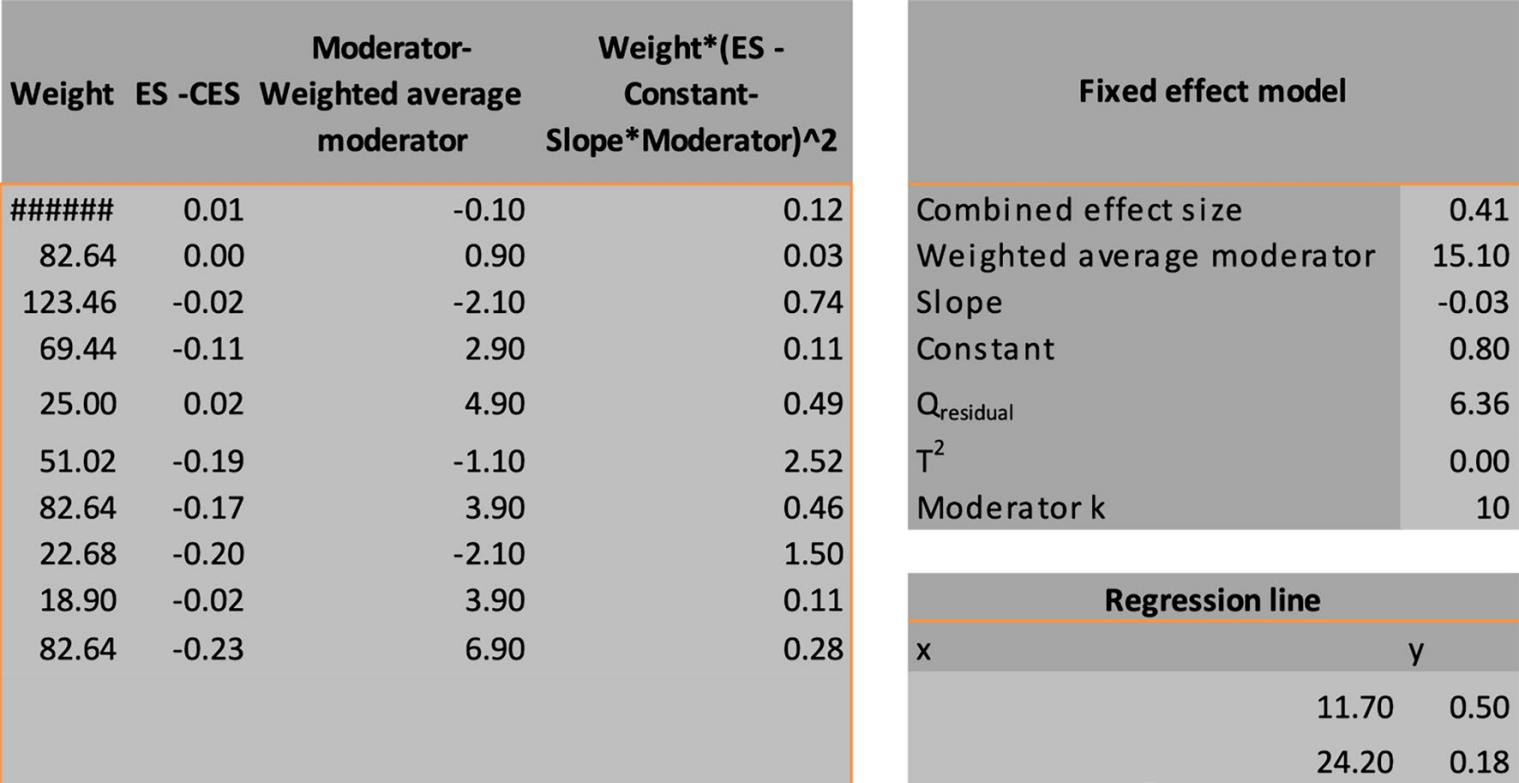
Regression of the moderator on effect size. Q: adjusted p value, T^2^: difference between the mean values of the two groups

A subgroup analysis of postoperative visual acuity was conducted using a fixed-effects model because there was no significant heterogeneity (I^2^ = 0%). The six-month follow-up durations were divided into three and six months to assess the functional and anatomical outcomes of idiopathic and refractory MH surgeries, respectively. Five studies with a follow-up duration of three months were pooled for the first subgroup analysis. The analysis indicated that the difference in postoperative visual acuity at three months was significantly greater in the inverted ILM flap group for idiopathic patients than in the other treatment groups for refractory patients (WMD = − 0.03; 95% CI = − 0.22, − 0.07; Fig. 10). Five studies with a follow-up duration of six months were pooled for the second subgroup analysis. The analysis did not reveal any significant difference between the groups at six months (WMD = − 0.08; 95% CI = − 0.19, 0.03; p = 0.002).

## Discussion

The management and pathogenesis of MHs, which are idiopathic full-thickness retinal defects, remain controversial [4, 13]. However, the combined application of vitrectomy and adjuvant therapies, such as ILM techniques, improves the closure rate and has become the standard treatment method for MH [13]. Moreover, there is a dearth of literature on the effectiveness of ILM surgical techniques for large idiopathic and refractory MH management, especially from the perspective of the MH closure rate and postoperative visual acuity [10]. To address this gap, a meta-analysis involving 23 studies was conducted with a focus on four ILM surgical techniques. These techniques include classical ILM peeling, an inverted ILM flap, ART, and ILM insertion.

The present meta-analysis of 23 RCTs reported positive and effective treatment outcomes with classic ILM peeling, inverted ILM flaps, ART, and ILM insertion. Thus, the inverted ILM flap technique, although associated with improved postoperative vision at 3 months but not at 6 months may be preferred over other techniques due to improved anatomical closure rates, which may confer some long-term benefits. However, this finding needs to be confirmed with long-term follow-up. The findings of the current meta-analysis were consistent with those reported in the literature. For instance, Michalewska et al. applied the inverted ILM flap technique to treat MHs with diameters > 400 µm and achieved a success rate of 98% in 50 patients included in the study. The study findings indicated that after surgery, all 50 patients achieved visual improvement and MH closure (*P* = 0.0027) [14]. The inverted ILM flap technique has been used mainly for idiopathic nonoperated MH patients and is considered safe and effective for repairing large, full-thickness MHs [11].

The results of the meta-analysis indicated that the inverted ILM flap technique was associated with significantly greater improvement in visual acuity than ILM peeling, ART, or ILM insertion. These findings are consistent with those reported in the literature, which indicates that the inverted ILM flap technique is associated with improved functional outcomes after surgical closure of large MHs [15]. This technique is reported to have a greater incidence of type 1 closure in large MHs than other treatment options, especially classic ILM peeling [16]. To minimize the limitations of classic ILM peeling, another modified procedure, the use of an inverted ILM flap technique was introduced. This procedure is reportedly safe and successful for managing large idiopathic MHs with effective outcomes [17]. However, various problems, such as misplaced flap loss in the cutter probe, have been observed with the inverted ILM flap technique [18]. The MIP technique was introduced to reduce flap-related complications and increase the anatomical closure rate [19], and the possibility of flap loss difficulties may be approached using the lens capsule, which is considered an alternative scaffold for facilitating MH closure with favorable and improved vision [16].

Vitrectomy with classical ILM peeling has been reported as the most successful surgical technique for MH treatment [15]. However, several changes in retinal structure and function have been observed because of the use of this ILM peeling technique [20]. For large MHs, the closure rate is usually low [21]. ART techniques have also been applied for refractory MH closure with anatomical improvements but disappointing or decreased functional results. Moreover, the combined application of autologous platelet concentrate, and ILM peeling has been shown to improve anatomical and functional outcomes in the management of chronic idiopathic and refractory MHs [16]. However, currently, the combination of vitrectomy with ILM peeling has reached a milestone as a widely used treatment method for MH, with a 93%–98% closure rate [5]. According to previous studies, the success of MH surgeries has increased gradually with the use of various manipulations and the upgrading of ILM surgical procedures in combination with surgical adjuncts [12, 15]. Patients with limited ILM may also have potential outcomes or MH closure with surgical options such as ARG, hAMG, AMP, or the creation of a distal ILM flap [3].

The short- and long-term visual efficacies of four surgical methods, namely, classic ILM peeling, inverted ILM flap, ART, and ILM insertion, were determined via subgroup analysis of postoperative visual acuity. The results indicated that the inverted ILM flap technique significantly improved vision at the 3 month follow-up compared with the other three techniques in patients with idiopathic MH. However, there was no statistically significant difference between the groups at the 6 month follow-up. This systematic review and meta-analysis revealed that the inverted ILM flap technique was effective at facilitating MH closure and significantly improved vision at the 3 month follow-up; however, there was no significant improvement in postoperative visual acuity at the 6 month follow-up or longer. The literature supports the findings of this meta–analysis by indicating that while the inverted ILM flap technique has a greater closure rate at the 3 month follow-up, its application does not result in better visual recovery during long-term follow-up [21].

Other MH treatment methods, such as ocriplasmin, have been used in the management of small- or medium-sized MHs with limited success [4]; however, according to a comparative study, the closure rate of MHs is greater after vitrectomy than after ocriplasmin [18]. 27-gauge vitrectomy has been suggested for use in combination with ILM peeling as a standard procedure for treating MH, as it results in considerable visual acuity improvements with few complications, such as PTMH formation, FTMH postoperative reopening or significant postoperative macular membrane formation [22, 23]. In MHs ≤ 400 µm and ≥ 400 µm, air tamponade and SF_6_ tamponade in combination with nonsupine have been used to achieve a high closure rate [24]. Several meta-analyses have suggested that visual benefits are observed in large MHs treated with the face-down posturing method but this posturing is considered unnecessary in smaller MHs; however, additional RCTs are needed to determine this benefit [25–28].

Several techniques have been applied to improve MH outcomes, especially in patients with large and refractory MHs such as enlarged or extended ILMs. However, no significant information was found to be included in this meta-analysis. Al Sabti et al. successfully achieved closure of two very large MHs, measuring 773 and 1147 μm, by enlarging the peeling of the ILM up to the arcades. Both eyes showed improvement in visual function after the surgery [29]. For refractory MHs that remain unresolved even after ILM removal with the help of dye, expanding the ILM-rhexis from the previous peel procedure may offer further advantages. Nevertheless, a preliminary investigation on the reoperation of refractory MHs that did not respond to initial PPV showed a reduced success rate in closing the MHs and a negative visual prognosis, even after undergoing secondary surgery [30]. Reoperation with this technique resulted in closure rates ranging from 46.7% to 68.9% in patients with refractory MHs. This involves enlarging the ILM peel up to the vascular arcade and the posterior fundus to release additional tangential traction on the MH [31, 32]. In most cases, the surgical approach with the enlarged ILM peeling technique closes the IMHs and restores vision with reduced visual distortion attributable to the reduction in asymmetric elongation of the foveal tissue. These outcomes suggest that patients who have previously undergone unsuccessful surgical attempts to treat idiopathic MH may benefit from an increase in the extent of ILM peeling. Surgical enlargement via ILM peeling closed the MHs and improved the logMAR BCVA in most patients [33].

The systematic review and meta-analysis conducted in the present study investigated the effects of ILM treatment on patients with MH worldwide and revealed the suitability and safety of ILM treatment methods as well as favorable and increased visual acuity in these patients. The risk of bias analysis revealed a low risk of bias in the studies considered, indicating the strongest evidence of bias in the studied domains, including the intervention groups, observed outputs, and result selection. This study will be helpful for surgeons treating MH with appropriate procedures and will provide novel insights into the improved application of treatment methods.

## Conclusion

This meta-analysis confirmed that the inverted ILM flap technique has a greater anatomical closure rate than classical ILM peeling, ART, or ILM insertion for idiopathic MH. In addition, this technique had better visual efficacy in the short-term follow-up than other MH treatment options. All the articles reported that the application of various ILM surgical techniques successfully transformed the untreatable history of MHs into better and more satisfactory morphological and functional outcomes with improved visual acuity. Based on these findings, it is plausible to conclude that the inverted ILM flap technique should be adopted as a routine and preferred procedure for the treatment of patients with large idiopathic MHs; in refractory MH, the present meta-analysis of 23 RCTs reported positive and effective treatment outcomes using ART with ABC, or MIP, followed by autologous ILM transplantation techniques, hAMG provides a high anatomical success with disappointing final vision. The present study provides clear insight into MH surgeries performed using ILM techniques and the observed visual acuity and anatomical closure rates, which can help clinicians choose accurate diagnostic and treatment methods for idiopathic and refractory MH surgeries to achieve better outcomes.

### Supplementary Information

Supplementary Material 1.

## Data Availability

The datasets used in this study have been included in the main text. Photographs and figures from this study may be released via a written application to the Photographic Laboratory and Clinical Archives Retina Department at the Oftalmologia Integral ABC Medical and Surgical Assistance Institution (Nonprofit Organization), Av. Paseo de las Palmas 735 suite 303, Lomas de Chapultepec, Mexico City 11000, Mexico and the corresponding author upon request.

## Abbreviations

ABC: Autologous blood clot
AMP: Amniotic membrane plug
ARG: Autologous retinal graft
ART: Autologous retinal transplant
BCVA: Best-corrected visual acuity
CI: Confidence interval
EZD: Ellipsoid zone density
FTMH: Full-thickness macular hole
FSIP: Fovea sparing internal limiting membrane peeling
FS-ILM: Fovea-sparing internal limiting membrane
hAMG: Human amniotic membrane graft
I^2^: Percentage of variation
ILM: Internal limiting membrane
IMH: Idiopathic macular hole
LL: Lower limit
MeSH: Medical subject headings
MIP: Multilayer internal limiting membrane plug
MH: Macular hole
OR: Odds ratio
PMID: PubMed identifier
PTMH: Partial-thickness macular hole
PICOS: Population, intervention, comparison, outcome, setting
PMID: PubMed identifier
PRISMA: Preferred Reporting Items for Systematic Reviews and Meta-Analyses
Q: Adjusted p value
QoL: Quality of life
RCT: Randomized clinical trial
R^2^: Coefficient of determination
RoB2: Risk-of-bias
RMH: Refractory macular hole
RPE: Retinal pigment epithelium
SE: Standard error
SF_6_: Sulfur hexafluoride
T: T value measures the size of the difference relative to the variation in sample data.
T^2^: Difference between the mean values of the two groups
UL: Upper limit
VA: Visual acuity
WMD: Weighted mean difference
