# Supplementary material for: Effect of internal limiting membrane surgical techniques on the idiopathic and refractory management of macular holes: a systematic review and meta-analysis
Source: Int J Retina Vitreous. 2024 Jun 21;10:44. doi: 10.1186/s40942-024-00564-2 (PMC11193206; doi:10.1186/s40942-024-00564-2)
Supplement: Supplementary file 1 — Supplementary Material 1. [file 40942_2024_564_MOESM1_ESM.doc]

**APPENDIX 1. Supplementary file**

**Search Strategy**

**MeSH Terminologies to be Used:**

1. Idiopathic macular hole AND Refractory macular hole AND Internal limiting membrane techniques AND surgery
2. Idiopathic macular hole AND Refractory macular hole AND Inverted flap internal limiting membrane removal techniques AND surgery
3. Idiopathic macular hole AND Refractory macular hole AND Lens capsule transplantation
4. Idiopathic macular hole AND Refractory macular hole AND multilayered inverted internal limiting membrane technique
5. Idiopathic macular hole AND Refractory macular hole AND Free autologous internal limiting membrane transplantation
6. Idiopathic macular hole AND Refractory macular hole AND Autologous neurosensorial retinal grafting
7. Idiopathic macular hole AND Refractory macular hole AND Amniotic membrane grafting technique
8. Idiopathic macular hole AND Refractory macular hole AND FSIP technique AND FS-ILM removal technique

**Search Databases to be Used:** PubMed, Ovid, Cochrane, GoogleScholar, Scopus, Embase

Articles were searched in publicly available online literature databases, including PubMed (<https://pubmed.ncbi.nlm.nih.gov/>), Scopus (<https://www.scopus.com/home.uri>), Ovid (<https://ovidsp.ovid.com/>), Cochrane (<https://www.cochranelibrary.com/>), Google Scholar (<https://scholar.google.com/>), and Embase (<https://www.elsevier.com/en-in/solutions/embase-biomedical-research>) using MeSH terminologies such as “Idiopathic macular hole AND refractory macular hole AND internal limiting membrane techniques AND surgery,” “Idiopathic macular hole AND refractory macular hole AND internal limiting membrane removal technique,” “Idiopathic macular hole AND refractory macular hole AND lens capsule transplantation,” “Idiopathic macular hole AND refractory macular hole AND inverted internal limiting membrane technique,” “Idiopathic macular hole AND refractory macular hole AND inverted internal limiting membrane technique,” “Idiopathic macular hole AND free autologous limiting membrane transplantation,” “Idiopathic macular hole AND refractory macular hole AND autologous neurosensorial retinal grafting,” “Idiopathic macular hole AND refractory macular hole AND amniotic membrane grafting technique,” and “Idiopathic macular hole AND refractory macular hole AND FSIP technique AND FS-ILM removal technique”.
